# *O data*, *where art thou?* Revolutionizing data sharing to advance our sustainability goals through smart chemical innovation

**DOI:** 10.1016/j.isci.2022.105256

**Published:** 2022-10-02

**Authors:** Jakub Kostal, Bryan W. Brooks, Christopher A. Smith, Geetesh Devineni

**Affiliations:** 1 Department of Chemistry, The George Washington University, Washington, DC, USA; 2 DOT Consulting LLC, Alexandria, VA, USA; 3 Department of Environmental Science, Center for Reservoir and Aquatic Systems Research, Institute of Biomedical Studies, Baylor University, Waco, TX, USA; 4 University of South Bohemia in České Budějovice, Faculty of Fisheries and Protection of Waters, South Bohemian Research Center of Aquaculture and Biodiversity of Hydrocenoses, Zátiší 728/II, CZ-389 25 Vodňany, Czech Republic; 5 Redshift Technologies Inc., New York, NY, USA

**Keywords:** Chemistry, Organic chemistry, Green chemistry, Toxicology, Environmental toxicology

## Abstract

Antiquated and inefficient data-sharing practices represent one of the key obstacles to advancing sustainability goals through green chemistry. To this end, we need to robustly link data on chemical impacts with new chemical design strategies, which requires the development of next-generation data-sharing platforms to harmonize both data and efforts. These decentralized and interactive programs should be structured as *live* ecosystems for data generation and exchange, inviting conversations about the reliability and relevance of information used to make decisions regarding chemical performance and safety.

## Graphical abstract

**Figure:**
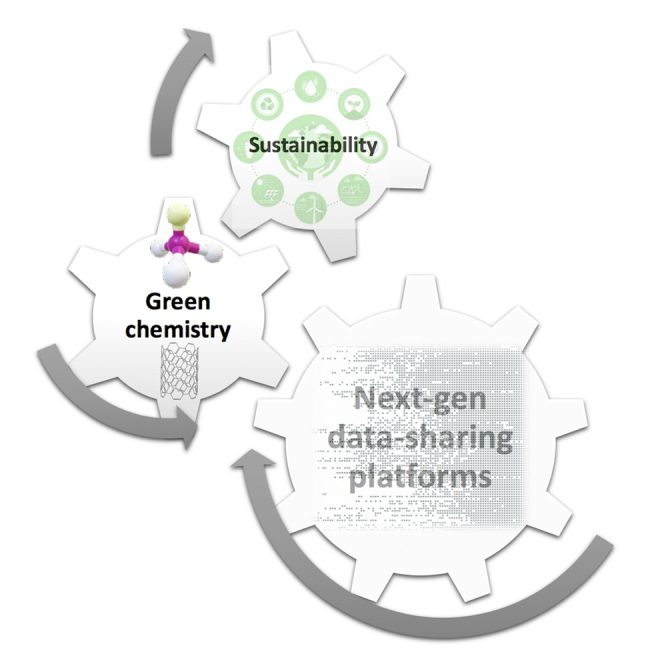

## Unintended consequences creating our shared “toxic debt”

Over the course of the 20^th^ century, we deliberately entangled our lives in chemistry on a previously unimaginable scale (Ilgen, 1983). This was best manifested by “Better Living Thru Chemistry”, DuPont’s advertising slogan. While its economic and societal benefits are widely embraced and clearly undeniable, chemistry has also done irreparable harm; DuPont’s production of per- and polyfluorinated substances (PFAS), which surfaced in 1999, provides an exemplar, as these “forever chemicals” are both persistent and toxic (Podder et al., 2021). Indeed, most chemicals on the market today were designed for their cost and performance, without consideration of environment, health and safety. Fortunately, observations of environmental damage spurred toxicological studies and subsequent development of environmental policies and regulatory activities over the past century (Dogo and Jaganjac, 2007), including for chemical classes such as PFAS (Brennan et al., 2021). However, attempts to manage risks to public health and the environmental have focused on reducing exposure (rather than mitigating hazard at the design stage), and so in this reactive approach, we have always played catch-up (Anastas and Warner, 1998), ignoring the true cost of the chemical revolution. For many persistent chemicals, this cost has become a loan rapidly accruing interest, which we (and our children) will have to pay off, a reality that is slowly dawning on society (Landrigan et al., 2018). For example, some 10%–20% of detected pesticides are stable transformation products, many originating from hazardous chemicals that were banned decades ago (Zhang, 2018). To bring this current reality full circle, the Centers for Disease Control and Prevention (CDC) estimates that most of us have PFAS in our blood,(CDC) and, while concentrations of the well-known actors (PFOS, PFOA, etc.) might be declining (Olsen et al., 2017), this is likely due to the use of (not necessarily safer but less-studied) alternatives (US EPA).

Revelations about the adverse health effects of lead, mercury, asbestos, PFAS, polychlorinated biphenyls (PCBs), and dioxins have demonstrated the need to examine chemicals rigorously before they are commercialized. Since 1950, over 140,000 new industrial chemicals have been synthesized, and of these, ca. 5,000 are high-volume substances with nearly universal human exposure (Landrigan et al., 2018). Furthermore, at least 300,000 chemicals and chemical mixtures have been registered for commercial use around the world (Wang et al., 2020). Yet, most of these chemicals have not been empirically tested for safety or toxicity, with disproportionate impact on low-income countries and marginalized communities (Landrigan et al., 2018). To engage this toxic debt, green chemistry emerged in the late 1990s (Anastas and Warner, 1998), promoting health and safety at the design stage of chemicals over unsustainable “reactive” testing (Matus et al., 2010). At the same time, toxicology has shifted its focus from animal studies to the ethically and economically favorable New Approach Methodologies (NAMs) to better support this new paradigm (Punt et al., 2020; Brooks et al., 2020; Kostal and Voutchkova-Kostal, 2020). These methods have yielded a wealth of mechanistic toxicological data, and, when paired together (e.g., alternative vertebrates and *in vitro* assays informing *in silico* modeling), NAMs have the potential to generate a wealth of knowledge that is fundamental for safer chemical design. Our challenge now is to radically improve the way we share scientific information to better integrate high-quality data into chemical design and avoid regrettable substitutions when replacing hazardous chemicals on the market. Only in doing so can we advance toward our shared goals of equitably distributed green and sustainable chemistry, where the development and use of chemicals and materials is environmentally benign and stems from manufacturing processes that conserve natural resources.

## Barriers to sharing data

There are approximately 80,000 chemists and 9,000 toxicologists in the United States alone, a curious statistic indeed (United States Bureau of Labor Statistics, 2022). These number are large and, given that chemists make chemicals and toxicologists determine their risk, clearly out of balance. Disciplinary priorities aside, the first glaring obstacle in using someone else’s data is our unawareness that data exist for specific needs, followed by differential data formats, archiving, accessibility, and quality reporting across disciplines (Brooks et al., 2021). These issues are magnified by the rapidly growing scientific community, as evidenced by recent increases in STEM graduates (Fry et al., 2021), and our collective productivity. Based on a recent STM (International Association of Scientific, Technical and Medical Publishers) report, there are over 33,000 active scholarly peer-reviewed English-language journals and another ca. 9,000 non-English-language journals, collectively publishing upward of 3 million articles each year (STM, 2018). In recent years, these numbers have grown annually by ca. 4% for articles and over 5% for journals (STM, 2018). Consequently, no person and no research entity can stay “up to date” with information generated globally, even within their respective field(s) (Slavik, 2009). While critical and systematic reviews can help alleviate informational overload, review articles are not free of personal biases. These can result in “cherry picking”, shaping what data are promoted vs. marginalized (or ignored completely), particularly when review methods are not documented. Furthermore, review publications are constrained by rigid boundaries within and between science fields, which were created by a system that rewards specialization and favors reductionist (vs. holistic) thinking (Bateman and Hess, 2015). Scientific “tunnel vision” decreases our awareness of data/knowledge landscapes, which is problematic when we are required to look outside our niche, and “translate” information from further afield in interdisciplinary engagements (Stirling, 2014).

Informational unawareness aside, barriers to data sharing can be erected deliberately when data sharing opposes personal interests and, as is often the case in industry, constraints imposed by the employer (e.g., due to intellectual property rights). In Academia, scientists may be reluctant to share data (and metadata in particular) if it was hard to obtain as they compete to innovate, publish, and fund their research (Walsh et al., 2007; Blumenthal et al., 2006; Miller, 2015). They may also be apprehensive of other investigators manipulating or exploiting their data for personal gain (Miller, 2015), or concerned that wide data accessibility invites a level of scrutiny that may not be desirable (Coudert, 2019). Here, we should note that many academics do share data, as it is increasingly expected by funders and publishers. Unfortunately, while their data are kept safe by university firewalls and complex authentication systems (Smith et al., 2005), researchers often receive little formal training on data management and secure *intentional* sharing of data (Tenopir et al., 2011). Funding agencies are attempting to remedy this issue (Tenopir et al., 2011); however, it is difficult to envision that we can overcome barriers of our respective normative cultures without data-sharing platforms. These systems can entice users with robust data standards and high levels of security (i.e., doing much of the legwork that peer-to-peer sharing resists), thus reaching broader communities faster.

Presently, at the platform level, current data sharing in the sciences relies on centralized databases, which collect publicly available information. Academic journals are such repositories, as are standalone, subject-dedicated databases; for toxicology and green chemistry applications, alttox.org provides a comprehensive list of these public and private tools (http://alttox.org/resource-center/databases/). The former suffers from the “tragedy of riches”, with too much unstructured and non-standardized data in disparate catalogs and documents (Slavik, 2009). In particular, we do not adequately enforce systematized inclusion of relevant details in peer-reviewed publications, which is needed to vet data, and is critical to fields with new emerging data streams (e.g., NAMs in toxicology). Characterization and quantification of underlying uncertainties is key to the use of existing data (Kostal et al., 2020); yet, it is rarely done, in large part because important metadata is missing from publications (Kostal and Voutchkova-Kostal, 2020). On the other hand, the main concern with specialized databases is that their structure, content, and (intended) use is decided solely by the developer. Furthermore, as many of these projects are funded by grants, data repositories run the risk of “evaporating” as these programs lose funding or are retired due to obsolescence (Miller, 2015). A particularly relevant example is the Carcinogenic Potency Project, which developed the Carcinogenic Potency Database between 1980 and 2005 (Fitzpatrick, 2008). While the data are still accessible, it is no longer updated or curated, leaving its quality in doubt.

At its core, a data-sharing platform sources information from individual efforts, and at the individual level, when *we* share data with others in laboratory meetings, national conferences, grant applications, manuscripts etc., scientific value is traded for traditional rewards of personal gain, such as promotion, reputation, and prestige (Murray, 2010). While the developer decides the scope and format of a database, they are limited by the level of disclosure that the shared data provide. To this end, data sourced indirectly (by scavenging the digital landscape) rarely include all relevant metadata and a detailed “user guide” that would interpret data for a broader audience of potential end-users (Miller, 2015). To offer an anecdote, in our past collaborative efforts in toxicology and green chemistry (Coish et al., 2018), miscommunication due to lack of understanding was initially a barrier before we became more aware of other disciplines and could “translate” our respective vernaculars. This represented a considerable undertaking but bore unique fruit in a robust, interdisciplinary framework for safer chemical design (Coish et al., 2018).

## The opportunity cost of data-sharing barriers

As hinted above, scientific progress and its resulting societal benefits increase when information is shared (Shapin, 2008). We should resist scientific “tunnel vision” and reductionist tendencies because environmental science and technology in general (Brooks et al., 2021), and chemical hazard and risk assessments in particular, call for multidisciplinary efforts, where combining data and knowledge from several fields is necessary (Kostal and Voutchkova-Kostal, 2020). By the same token, it is straightforward to make a case for green and sustainable chemistry needing extensive interdisciplinary collaboration (Constable, 2021). Crucially, interdisciplinary research, which hinges on mutual understanding, reciprocally beneficial cooperation, and effective data sharing, can achieve superior funding performance, both in terms of volume and long-term value (Sun et al., 2021).

Our recent collaboration with pharmaceutical chemists and toxicologists to improve predictive models for peptide couplers, a unique class of dermal sensitizers, is a timely example of the value gained by overcoming resistance to sharing prized data and information (Graham et al., 2022). In this case, mutual willingness of pharmaceutical companies and model developers to collaborate, where proprietary structures and expert knowledge were on the line, yielded better understanding of the underlying biochemistry; improved predictive models; and provided clues for the design of more benign future analogs (Graham et al., 2022). With intellectual property at stake, our collaboration was made possible by advances in data-sharing technologies (Farrall et al., 2021), which facilitated safe and secure data transfers to protect competitiveness and privacy of the involved parties. What this exercise showed was that while competitiveness may initially decrease the likelihood of scientific sharing, perceived conformity of the engaged parties to open-science practices and reciprocity in addition to gains of new commercial capabilities and avenues for business growth can increase sharing in practice (Haeussler, 2011).

The above example of effective peer-to-peer sharing does not circumvent the need for a data-sharing platform. Indeed, the outcomes of our study were eventually shared with the broader scientific community in a peer-reviewed publication (Graham et al., 2022). As noted previously, the lack of a universal standard for (meta) data sharing in journals limits further usability of said data. Using the ToxRTool to assess study reliability (Schneider et al., 2009), we recently carried out an exercise to scrutinize (published) experimental data that have been used for decades to train predictive models for skin permeability, an important route of exposure for many commercial chemicals. We found that ca. 20% of this set was misreported, and the majority of data were of insufficient quality to be regarded as reliable in model development. Nonetheless, tens of predictive models were developed and published using this data over the past 2–3 decades, showing that without adequate data sharing, which would allow proper vetting of data, we are propagating uncertainties and “building on sand”. In toxicology and green chemistry, the problem can be exacerbated by modern statistical tools (i.e., those using machine learning and artificial intelligence), where different data types often need to be combined to generate large-enough training sets that cannot be curated manually (Kostal and Voutchkova-Kostal, 2020; Cherkasov et al., 2014).

## Setting up the goalposts

A solution to both incentivization of data sharing (in the modern vernacular) and a robust system to support cross-disciplinary research efforts in green and sustainable chemistry can be found in next-generation data-sharing platforms (DSPs). We envision these DSPs as decentralized ecosystems that support decision-making of its users (vs. mere data collection). To this end, DSPs should enable live user interactions in data exchange, generation, and analysis, and promote multi-tenant collaboration in a common core (i.e., “gated” collaboration across organizations, where data security is paramount). Such framework would mitigate issues related to different data-sharing requirements and restrictions across disciplines, which is important to both hazard assessment and systems-based development of new chemicals and materials (Constable, 2021), as briefly illustrated by our aforementioned case study on peptide couplers.

The cornerstone of a DSP ecosystem should be an integrated and searchable knowledge base that is defensible and repeatable (i.e., fully transparent and of high-quality data). It should incorporate data ranking tools that allow any user to store, develop, and use environmental fate and safety assessments to foster the development of chemical alternatives safer for public health and the environment. DSPs should be designed as flexible knowledge management solutions that integrate both predicted and experimental data sources, with the goal to leverage data sharing, collaboration, and customization so as to address the diverse needs of the toxicology and green chemistry communities. As noted above, the knowledge base would rely on a multi-tenant data structure with a shared application layer, which will allow tenant flexibility with regards to scalability, cost, complexity, and customization, and attract users across academia, industry, and government. Elements of such architecture exist, such as in Microsoft 365 for business tenants.

Under the hood, DSPs would provide answers to user questions and support individualized operations by transforming critical data into visual “story books”, designed to illustrate the compelling relationships between data and specific user needs. These story books would facilitate a personalized access to workflows in the system, giving users the ability to visualize information based on their roles (e.g., researcher, regulator, consumer, or project administrator). For example, while some users will employ a DSP dashboard to track and summarize chemical designs, manage starting materials, perform chemical read-across, or track molecular substitutes, others may use the system to analyze risks, manage supply chain, generate operational impact trends, or follow chemical predictor performance. On the back of predictive algorithms, such as AI or machine learning (ML), social media platforms have pioneered these functionalities to tailor user content. Facebook wall is an example of how data are effectively turned into a customized storyline for the user based on user characteristics and past behavior on the site.

Incentivizing “buy-in” to overcome data-sharing resistance, especially when data are sourced directly and need to conform to a high standard/disclosure, can be achieved through *data bartering*. Here, access to existing data can be offered in exchange for user’s own data or for services such as data generation, curation, or analytics within DSPs. To draw a parallel, consumers of Facebook or Google barter their personal data for digital services, such as messaging systems or map tracking. “Crowdsourcing” data manipulation activities on DSPs would further aid in data interpretation and in recognizing and alleviating data quality-related pain points (Beck et al., 2022), mimicking benefits of this approach elsewhere (Zhen et al., 2021). Data quality is an important consideration in the development of sustainability claims and in providing feedback for research activities, production efficiency, and environmental-quality improvements within the supply chain. In an effort to support the best decisions possible within DSPs, users would be tasked to incorporate confidence/uncertainty metrics, which increase transparency and recognize the variable nature of living systems that are being represented by the data. Provided sufficient metadata by the user, these calculations could also be performed on the backend, using existing approaches (Kostal et al., 2020; Rathman et al., 2018; Park et al., 2014; Yang et al., 2013).

The hurdle of data-format standardization, which is recognized as one of the key barriers to data sharing (van Panhuis et al., 2014), can be minimized by integration of data analytics methods based on ML. While these approaches may have differential mechanistic potential to robustly inform molecular initiation events and adverse outcomes (Alves et al., 2019), and appear even less applicable to *rational (and defensible)* design of safer chemicals (Kostal and Voutchkova-Kostal, 2020), their power in mining toxicological data and facilitating both data systematization and categorization is evident (Cheng et al., 2021). ML can provide other exciting features for future DSPs, such as tracking (and reconciling) user discord within the platform as an indirect metric of data uncertainty, in effect turning the platform into a “scientific town square”, akin to social media platforms (Watercutter, 2022). This level of monitoring and subsequent data and user-behavior analytics would offer a more comprehensive report on the field’s *status quo*, reflecting the diversity of metadata and assessment practices. ML has been used successfully for similar purposes by companies such as Facebook or Google to customize ranking for news feeds, speech recognition, and translations; and track website visits, geographic data, and time spent on tools/features, measuring their popularity (Song et al., 2018; Claussen et al., 2013). Data can be in turn leveraged to increase user engagement within the platform; to make its content more relevant; and to expand the platform structure in directions aligned with users’ needs and interests. In the end, knowing that data will be part of a grander scheme may encourage scientists to focus more on the quality, usability, and integrity of their data.

## Looking ahead

It is difficult to envision that the proposed effort could be successfully tackled by a single lab or even by the typical-sized collaborative. Buy-ins from key stakeholders in academia, industry, and the government are essential to developing a pilot platform that would attract sufficiently large user base, and thus attain sustained growth and the proposed data-sharing paradigm shift. Furthermore, these efforts cannot depend on funding that may run out (Miller, 2015). Ideally, funding agencies such as the National Institutes of Health and the National Science Foundation, which already require data management plans from its grantees, could take up the proverbial baton. Scientific societies could also offer leadership, as they would undeniably benefit from a combined, sharable, and robust database standard for their diverse activities. With appropriate leadership from organizations that provide research funding and facilitate its outcomes, research organizations and researchers themselves would follow suit. While the vision and blueprints could be created “in house”, i.e., by a panel of key toxicology and green chemistry stakeholders, in the end it is the private (tech) sector that has the necessary knowhow and could develop and maintain a DSP of this magnitude and complexity at the behest of the scientific community. This trajectory is in line with recent initiatives that seek to improve the *status quo* in data sharing (Kozlov, 2022; Cao et al., 2016), increasing commitment to robust data-sharing practices from scientific journals and funding agencies (Tenopir et al., 2011; Miller, 2014), and calls for global changes in science policy to tackle chemical pollution (Brack et al., 2022).

In the absence of leadership with the vision and ability to deliver on this charge, scientists are bound to continue to struggle in inefficient relationships with their own data and with each other, particularly as we aim to increase innovation through interdisciplinary research by embracing systems-based approaches (Constable, 2021). We will continue to compete in a way that is counterproductive to our collective progress (Fang and Casadevall, 2015), and chemical development and management will continue to be a Sisyphean game of whac-a-mole, a reality that requires acceleration of safer chemicals and sustainable chemistry. Many may find solace that though we are moving slowly on existential issues such as global chemical pollution or climate change (Brack et al., 2022; Lim, 2021), we are moving in the right direction. A few recognize that such sentiment is profoundly problematic—unless the rate of positive change outcompetes the rate of negative change, for which the wheels were set in motion a long time ago, we will not succeed in protecting public health and the environment and achieving a sustainable and equitable world. Now is the time to start pulling on the same rope and sharing data effectively, so that we can realize a shared future for all life.

### Limitations of the study

The present perspective is limited in its assessment of data-sharing practices by surveyed literature and professional experiences of the authors. While all limits are self-imposed (Icarus), they are ultimately unavoidable.

## References

[bib47] Alves V.M., Borba J., Capuzzi S.J., Muratov E., Andrade C.H., Rusyn I., Tropsha A. (2019). Oy Vey! A comment on "machine learning of toxicological big data enables read-across structure activity relationships outperforming animal test reproducibility. Toxicol. Sci..

[bib5] Anastas P.T., Warner J.C. (1998).

[bib61] STM (2018).

[bib58] CDC PFAS in the U.S. Population. https://www.atsdr.cdc.gov/pfas/health-effects/us-population.html

[bib21] Bateman T.S., Hess A.M. (2015). Different personal propensities among scientists relate to deeper vs. broader knowledge contributions. Proc. Natl. Acad. Sci. USA.

[bib41] Beck S., Brasseur T.M., Poetz M., Sauermann H. (2022). Crowdsourcing research questions in science. Res. Policy.

[bib24] Blumenthal D., Campbell E.G., Gokhale M., Yucel R., Clarridge B., Hilgartner S., Holtzman N.A. (2006). Data withholding in genetics and the other life sciences: prevalences and predictors. Acad. Med..

[bib55] Brack W., Barcelo Culleres D., Boxall A.B.A., Budzinski H., Castiglioni S., Covaci A., Dulio V., Escher B.I., Fantke P., Kandie F. (2022). One planet: one health. A call to support the initiative on a global science–policy body on chemicals and waste. Environ. Sci. Eur..

[bib4] Brennan N.M., Evans A.T., Fritz M.K., Peak S.A., von Holst H.E. (2021). Trends in the regulation of per- and polyfluoroalkyl substances (PFAS): a scoping review. Int. J. Environ. Res. Public Health.

[bib14] Brooks B.W., Sabo-Attwood T., Choi K., Kim S., Kostal J., LaLone C.A., Langan L.M., Margiotta-Casaluci L., You J., Zhang X. (2020). Toxicology advances for 21st century chemical pollution. One Earth.

[bib17] Brooks B.W., Arnold W.A., Boehm A.B., Martin J.W., Mihelcic J.R., Schlenk D., Wang S. (2021). Quantity, quality, and accessibility: big data collection, analysis, and synthesis in environmental science and technology. Environ. Sci. Technol. Lett..

[bib53] Cao Y., Jones C., Cuevas-Vicenttin V., Jones M.B., Ludascher B., McPhillips T., Missier P., Schwalm C., Slaughter P., Vieglais D. (2016). DataONE: a data federation with provenance support. Lect Notes Comput Sc.

[bib48] Cheng F., Li H., Brooks B.W., You J. (2021). Signposts for aquatic toxicity evaluation in China: text mining using event-driven taxonomy within and among regions. Environ. Sci. Technol..

[bib40] Cherkasov A., Muratov E.N., Fourches D., Varnek A., Baskin I.I., Cronin M., Dearden J., Gramatica P., Martin Y.C., Todeschini R. (2014). QSAR modeling: where have you been? Where are you going to?. J. Med. Chem..

[bib51] Claussen J., Kretschmer T., Mayrhofer P. (2013). The effects of rewarding user engagement: the case of Facebook apps. Inf. Syst. Res..

[bib32] Coish P., Brooks B.W., Gallagher E.P., Mills M., Kavanagh T.J., Simcox N., Lasker G.A., Botta D., Schmuck S.C., Voutchkova-Kostal A. (2018). The molecular design research network. Toxicol. Sci..

[bib34] Constable D.J.C. (2021). Green and sustainable chemistry - the case for a systems-based, interdisciplinary approach. iScience.

[bib26] Coudert F.X. (2019). Correcting the scientific record: retraction practices in chemistry and materials science (vol 31, pg 3593, 2019). Chem. Mater..

[bib3] Dogo H., Jaganjac A. (2007). Integrating the principles of sustainable development into public policy oriented curricula and syllabi. Annals of Daaam for 2007 & Proceedings of the 18th International Daaam Symposium.

[bib10] US EPA Human Health Toxicity Assessment for GenX Chemicals. https://epi.dph.ncdhhs.gov/oee/pfas/GenXToxicityAssess-Factsheet-WEB-122221.pdf.

[bib56] Fang F.C., Casadevall A. (2015). Competitive science: is competition ruining science?. Infect. Immun..

[bib37] Farrall F., Mittal N., Narra C., Tello J. (2021).

[bib30] Fitzpatrick R.B. (2008). CPDB: carcinogenic potency database. Med. Ref. Serv. Q..

[bib18] Fry R., Kennedy B., Funk C. (2021).

[bib36] Graham J.C., Trejo-Martin A., Chilton M.L., Kostal J., Bercu J., Beutner G.L., Bruen U.S., Dolan D.G., Gomez S., Hillegass J. (2022). Chemical Research in Toxicology.

[bib38] Haeussler C. (2011). Information-sharing in academia and the industry: a comparative study. Res. Policy.

[bib59] Ilgen T.L. (1983). Better living through chemistry - the chemical-industry in the world-economy. Int. Organ..

[bib15] Kostal J., Voutchkova-Kostal A. (2020). Going all in: a strategic investment in in silico toxicology. Chem. Res. Toxicol..

[bib29] Kostal J., Plugge H., Raderman W. (2020). Quantifying uncertainty in ecotoxicological risk assessment: MUST, a modular uncertainty scoring tool. Environ. Sci. Technol..

[bib52] Kozlov M. (2022). Nih issues a seismic mandate: share data publicly. Nature.

[bib6] Landrigan P.J., Fuller R., Acosta N.J.R., Adeyi O., Arnold R., Basu N.N., Baldé A.B., Bertollini R., Bose-O'Reilly S., Boufford J.I. (2018). The Lancet Commission on pollution and health. Lancet.

[bib57] Lim X.Z. (2021). Scientists call for IPCC-like group on chemical pollution. Chem. Eng. News.

[bib12] Matus K.J.M., Zimmerman J.B., Beach E. (2010). A proactive approach to toxic chemicals: moving green chemistry beyond alternatives in the "safe chemicals act of 2010". Environ. Sci. Technol..

[bib54] Miller G.W. (2014). Improving reproducibility in toxicology. Toxicol. Sci..

[bib25] Miller G.W. (2015). Data sharing in toxicology: beyond show and tell. Toxicol. Sci..

[bib31] Murray F. (2010). The oncomouse that roared: hybrid exchange strategies as a source of distinction at the boundary of overlapping institutions. Am. J. Sociol..

[bib9] Olsen G.W., Mair D.C., Lange C.C., Harrington L.M., Church T.R., Goldberg C.L., Herron R.M., Hanna H., Nobiletti J.B., Rios J.A. (2017). Per- and polyfluoroalkyl substances (PFAS) in American Red Cross adult blood donors, 2000-2015. Environ. Res..

[bib44] Park S.J., Ogunseitan O.A., Lejano R.P. (2014). Dempster-shafer theory applied to regulatory decision process for selecting safer alternatives to toxic chemicals in consumer products. Integr. Environ. Assess. Manag..

[bib60] Podder A., Sadmani A.H.M.A., Reinhart D., Chang N.B., Goel R. (2021). Per and poly-fluoroalkyl substances (PFAS) as a contaminant of emerging concern in surface water: a transboundary review of their occurrences and toxicity effects. J. Hazard Mater..

[bib13] Punt A., Bouwmeester H., Blaauboer B.J., Coecke S., Hakkert B., Hendriks D.F.G., Jennings P., Kramer N.I., Neuhoff S., Masereeuw R. (2020). New approach methodologies (NAMs) for human-relevant biokinetics predictions: meeting the paradigm shift in toxicology towards an animal-free chemical risk assessment. Altex-Altern Anim Ex.

[bib43] Rathman J.F., Yang C., Zhou H. (2018). Dempster-Shafer theory for combining in silico evidence and estimating uncertainty in chemical risk assessment. Comput. Toxicol..

[bib39] Schneider K., Schwarz M., Burkholder I., Kopp-Schneider A., Edler L., Kinsner-Ovaskainen A., Hartung T., Hoffmann S. (2009). ToxRTool", a new tool to assess the reliability of toxicological data. Toxicol. Lett..

[bib33] Shapin S. (2008).

[bib20] Slavik R.S. (2009). Information overload: we need to improve the signal-to-noise ratio. Can. J. Hosp. Pharm..

[bib27] Smith A., Greenbaum D., Douglas S.M., Long M., Gerstein M. (2005). Network security and data integrity in academia: an assessment and a proposal for large-scale archiving. Genome Biol..

[bib50] Song M.J., Ward J., Choi F., Nikoo M., Frank A., Shams F., Tabi K., Vigo D., Krausz M. (2018). A process evaluation of a web-based mental health portal (WalkAlong) using Google analytics. JMIR Ment. Health.

[bib22] Stirling A. (2014). https://www.theguardian.com/science/political-science/2014/jun/11/science-policy-research-silos-interdisciplinarity.

[bib35] Sun Y., Livan G., Ma A., Latora V. (2021). Interdisciplinary researchers attain better long-term funding performance. Commun. Phys..

[bib28] Tenopir C., Allard S., Douglass K., Aydinoglu A.U., Wu L., Read E., Manoff M., Frame M. (2011). Data sharing by scientists: practices and perceptions. PLoS One.

[bib62] United States Bureau of Labor Statistics, Occupational Employment and Wage Statistics (visited May 10, 2022).

[bib46] van Panhuis W.G., Paul P., Emerson C., Grefenstette J., Wilder R., Herbst A.J., Heymann D., Burke D.S. (2014). A systematic review of barriers to data sharing in public health. BMC Publ. Health.

[bib23] Walsh J.P., Cohen W.M., Cho C. (2007). Where excludability matters: material versus intellectual property in academic biomedical research. Res. Policy.

[bib11] Wang Z., Walker G.W., Muir D.C.G., Nagatani-Yoshida K. (2020). Toward a global understanding of chemical pollution: a first comprehensive analysis of national and regional chemical inventories. Environ. Sci. Technol..

[bib49] Watercutter A. (2022).

[bib45] Yang L., Neagu D., Cronin M.T.D., Hewitt M., Enoch S.J., Madden J.C., Przybylak K. (2013). Towards a fuzzy expert system on toxicological data quality assessment. Mol. Inform..

[bib7] Zhang W.J. (2018). Global pesticide use: profile, trend, cost/benefit and more. Proceedings of the International Academy of Ecology and Environmental Sciences.

[bib42] Zhen Y., Khan A., Nazir S., Huiqi Z., Alharbi A., Khan S. (2021). Crowdsourcing usage, task assignment methods, and crowdsourcing platforms: a systematic literature review. J. Softw. Evol. Proc..

